# The Pathogenesis and Treatment of Cardiovascular Autonomic Dysfunction in Parkinson’s Disease: What We Know and Where to Go

**DOI:** 10.14336/AD.2021.0214

**Published:** 2021-10-01

**Authors:** Shuzhen Zhu, Hualing Li, Xiaoyan Xu, Yuqi Luo, Bin Deng, Xingfang Guo, Yang Guo, Wucheng Yang, Xiaobo Wei, Qing Wang

**Affiliations:** Department of Neurology, Zhujiang Hospital, Southern Medical University, Guangzhou, Guangdong, China

**Keywords:** Parkinson’s disease, cardiovascular autonomic dysfunction, orthostatic hypotension, postprandial hypotension, supine hypertension

## Abstract

Cardiovascular autonomic dysfunctions (CAD) are prevalent in Parkinson’s disease (PD). It contributes to the development of cognitive dysfunction, falls and even mortality. Significant progress has been achieved in the last decade. However, the underlying mechanisms and effective treatments for CAD have not been established yet. This review aims to help clinicians to better understand the pathogenesis and therapeutic strategies. The literatures about CAD in patients with PD were reviewed. References for this review were identified by searches of PubMed between 1972 and March 2021, with the search term “cardiovascular autonomic dysfunctions, postural hypotension, orthostatic hypotension (OH), supine hypertension (SH), postprandial hypotension, and nondipping”. The pathogenesis, including the neurogenic and non-neurogenic mechanisms, and the current pharmaceutical and non-pharmaceutical treatment for CAD, were analyzed. CAD mainly includes four aspects, which are OH, SH, postprandial hypotension and nondipping, among them, OH is the main component. Both non-neurogenic and neurogenic mechanisms are involved in CAD. Failure of the baroreflex circulate, which includes the lesions at the afferent, efferent or central components, is an important pathogenesis of CAD. Both non-pharmacological and pharmacological treatment alleviate CAD-related symptoms by acting on the baroreflex reflex circulate. However, pharmacological strategy has the limitation of failing to enhance baroreflex sensitivity and life quality. Novel OH treatment drugs, such as pyridostigmine and atomoxetine, can effectively improve OH-related symptoms via enhancing residual sympathetic tone, without adverse reactions of supine hypertension. Baroreflex impairment is a crucial pathological mechanism associated with CAD in PD. Currently, non-pharmacological strategy was the preferred option for its advantage of enhancing baroreflex sensitivity. Pharmacological treatment is a second-line option. Therefore, to find drugs that can enhance baroreflex sensitivity, especially via acting on its central components, is urgently needed in the scientific research and clinical practice.

Parkinson’s disease (PD) patients are suffered by both motor and non-motor symptoms [1-3]. Cardiovascular autonomic dysfunction (CAD), which acts as a part of non-motor symptom spectrums, is composed by a heterogeneous group of diseases complexes including orthostatic hypotension (OH), postprandial hypotension, supine hypertension and nondipping. CAD is prevalent in PD patients and severely affects their life quality. However, few attentions have been paid on it. So far neither clear pathogenesis nor effective treatment has been established. The current study reviewed the pathogenesis of CAD, including neurogenic and non-neurogenic mechanisms and summarized the current pharmaceutical and non- pharmaceutical treatment with a discussion of future potential therapies.

## 1.Orthostatic hypotension

### 1.1 Summary

Orthostatic hypotension (OH) is a physical finding defined as a reduction of either a systolic blood pressure (BP) decreases of at least 20 mm Hg or a diastolic blood pressure decrease of at least 10 mm Hg within 3 minutes of an upright tilt with at least 60°or standing time [4, 5]. OH is prevalent among people with PD, which can occur in up to 14% of untreated de novo PD patients and about 30-78% of total PD patients [6-9]. Among them, about 16 to 20% of PD patients have symptomatic OH [10, 11]. The symptoms of OH are mainly caused by insufficient perfusion of target organs [12, 13]. For example, patients with cerebral hypoperfusion may show light-headedness, dizziness, vision loss, pre-syncope, or even syncope [14-16], whereas, patients with hypoperfusion within lung area may result in “coat hanger” pain or orthostatic dyspnea [17, 18]. Notably, sometimes OH is asymptomatic and is often ignored by clinical doctors. OH in PD has been considered to be a risk factor for cognitive decline, frequent falls [6], cardiovascular events[19, 20] and mortality [21, 22]. Although OH has seriously affected the quality of life and even life span of PD patients, there is no effective treatment yet partly due to unclear pathogenesis. Therefore, in this study, we focus on the review of the pathogenesis of OH, in order to seek effective therapeutic clues.

### 1.2 Pathogenetic mechanisms

**Figure 1.**
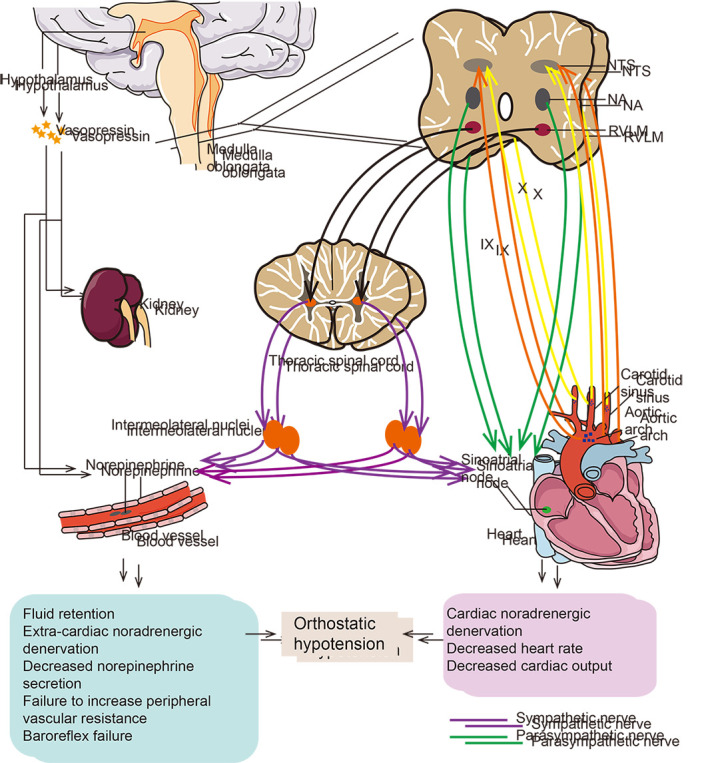
Triple whammy possible mechanisms of OH in PD. This figure describes the three mechanisms of neurogenic orthostatic hypotension. NOH in PD seems to reflect a “triple whammy” of cardiac and extra-cardiac noradrenergic denervation. Decreased BP stimulates arterial baroreceptors including carotid sinus, aortic arch and great vessels leading to a compensatory baroreflex response. Baroreceptor impulses are transmitted to the nucleus of the tractus solitarius (NTS) in the dorsomedial medulla along the afferent fibers, — the glossopharyngeal and vagus nerves. This is an inhibitory afferent fiber, that reduces impulse transmission to the NTS, which transmits signals to the nucleus ambiguous (NA), and the vagus nerve input from the nucleus ambiguous is reduced. At the same time, the NTS transmits signals to the rostral ventrolateral medulla connecting with the intermediolateral cell column of the thoracic spinal cord, and the sympathetic efferent nerve activity increases from the intermediolateral cell column of the thoracic spinal cord. Norepinephrine is the dominant neurotransmitter in the sympathetic efferent limb. Norepinephrine is the main neurotransmitter of sympathetic efferent limbs. The rapid increase of norepinephrine in response to standing, and an important pathophysiological mechanism of orthostatic hypotension in PD patients is its impaired response. NOH manifests with failure of baroreflex systems with or without sympathetic noradrenergic denervation. As a response to the continuous decrease of BP, the release of vasopressin is mediated by the projection of the A1 noradrenergic cell population in the ventrolateral medulla. The vasopressin-producing neurons in the magnocellular portion of the paraventricular nucleus and the supraoptic nucleus of the hypothalamus will be activated by this projection, resulting in increased HR, cardiac output and vasoconstriction. Cardiac and extra-cardiac noradrenergic denervation, baroreflex failure,and the decrease of noradrenaline release are also associated with ageing. Cardiac noradrenergic denervation can decrease heart rate and cardiac output. Extra-cardiac sympathetic denervation leads to a decrease in the release of norepinephrine, resulting in decreased peripheral vasoconstriction, reduced return heart blood volume, and eventual decreased BP.

The mechanisms underlying the development of OH in PD remain largely elusive. At present, the pathophysiological mechanisms are mainly composed by two parts: non-neurogenic and neurogenic parts. Non-neurogenic mechanisms include reduced intravascular volume, cardiac impairment and drug-induced hypotension. Neurologic mechanisms included cardiac and extra-cardiac noradrenergic denervation accompanied with impaired baroreflexes, which were called the “triple whammy” (Fig. 1) [5, 23].

#### 1.2.1 Non-neurogenic mechanisms

##### 1.2.1.1 The decrease of circulating blood volume

The decrease of effective circulating blood volume is one of the causes of OH in PD patients. Dysphagia, which is a common situation in advanced PD patients, is closely associated with insufficient fluid and poor nutrition intake, decline of intravascular blood volume and occurrence of OH [24-28].

##### 1.2.1.2 Cardiac dysfunction

Cardiac dysfunction, especially heart failure, was frequently found in PD patients and considered to be a risk factor for OH [29]. The prevalence of heart failure in elderly PD patients was almost 2.27 times that of non-PD patients (19.4% versus 8.7%), even after adjusting for stroke and possible vascular parkinsonism[30]. One the one hand, the deposition of Lewy bodies in the heart of PD patients may partly explain the underlying mechanism [31, 32]. On the other hand, the application of pramipexole is also considered to increase the risk of heart failure [33].

##### 1.2.1.3 Drug-induced hypotension

Antiparkinsonian drugs had long been listed as main suspects for OH in PD. The suspected anti parkinsonism drugs contain Levodopa and dopamine receptor agonists such as pramipexole, ropinirole and rotigotine. Antiparkinsonian drugs with the possibility to cause or exacerbate OH were summarized in Table 1.

Levodopa: Levodopa is one of the most effective symptomatic treatment options for Parkinsonism with a favorable safety and tolerability profile. However, it was found to be possibly responsible for postural hypotension. Kondo, M. reported that a case of Parkinson’s disease showed orthostatic hypotension due to levodopa administration with a dose of 500mg per day [34]. However, study from Mehagnoul-Schipper, D. J. showed that therapy with 125-mg b.i.d. doses of levodopa did not significantly aggravate orthostatic hypotension in older PD patients (age range from 66 to 84 years) [35]. Coincidentally, another study form Jost, W. H drew a similar conclusion. This study examined 99 PD patients with a mean age of 74 years. The duration of the disease was on average seven years. The drop in blood pressure after orthostasis was 43.75 mmHg in levodopa group and 45.46 mmHg in non- levodopa medication group, respectively. No significant statistical difference was found between these two groups [36]. In general, whether levodopa will lead to orthostatic hypotension is still controversial and need to be further confirmed.

**Table 1:** Representative studies on antiparkinsonian drugs -related orthostatic hypotension.

Drugs	Regimens	Type of clinical research	Sample characteristics	Result (mean±SD)	Conclusion	Author (year)
Levodopa	200mg/d * 10 d	Case report	Sample size: N=1 Gender: female; Age:70 y DD: 3 y	Systolic BP decline from 110-120 to 90 mmHg; OH	Controversial Need to be confirmed	Kondo, M. et al (2000)^1^
	62.5mg bid * 1w; 125-mg b.i.d. * 1w;	Double-blind, randomized, two-treatment cross-over trial	Sample size: N=17 (PD); N=17 (HC); Gender: N/A Age: 74.3 ± 0.9 y DD: N/A	OH was infrequently found in Parkinsonian patients (13%) and healthy subjects (6%) ; No significant difference.		Mehagnoul-Schipper, et al (2001)^2^
	Not mentioned	Prospective observational research	Sample size: N=99 Gender: 72 male, 27 females; Age: 74±7.8y; (range 52-88 y). DD: 7 ± 4.4 y (range 0-19 y)	The drop in blood pressure after orthostasis without levodopa medication reached on average 45.46 mmHg (SD = 23.76; SEM = 2.39), and the average drop after levodopa medication was 43.75 (SD = 17.88; SEM = 1.8). No significant difference.		Jost, W. H. et al (2020)^3^
DAs Pramipexole	1.5mg/d* 6-15 m	Double-blind, randomized, Controlled trail	Sample size: N=261 Gender: N/A Age: N/A DD: early stage, N/A	2 patients developed drug-related postural hypotension	Need to be confirmed	Schapira, A. H. (2013)^4^ On Underlying Disease (PROUD) Hubble, J. P. (1995)^5^
	An ascending dose of 4.5 mg/day*9w	Multicenter Study Randomized Controlled Trial	Sample size: N=55 Gender: N/A Age: N/A DD: early stage, N/A	All subjects in both the pramipexole and the placebo groups experienced one or more episodes of asymptomatic OH and there was no significant difference between the pramipexole and the placebo groups		
Ropinirole		Observational research	Sample size: N=50 Gender: 31 Female, 10 males Age: 61.4±4.3 y DD: early stage, N/A	One female patient was reported to have OH after the administration of ropinirole	Need to be confirmed	Titlic, M. (2008 )^6^
	9 mg/d*12w	Multi-center, randomized, double-blind, parallel-group trial	Sample size: N=221 Gender: N/A Age: N/A DD: early stage, N/A	20.34% cases were found with OH after 12 weeks’ treatment with ropinirole		Li, Shu-hua (2013)^7^
Rotigotine	8mg/24h * 8 w + levodopa and oral DA (pramipexole </=1.5 mg/day, ropinirole </=6.0 mg/d)	Open label, Clinical Trial, Phase III Observational research	Sample size: N=90 Gender: 43 Female, 47male Age: 61.3 ± 9.3 y DD: Longer than 3 years’ duration	9 (10%) was reported with OH	Need to be confirmed	Kim, Jong-Min (2015) ^8^
	Different dosage from 2-8mg/24h	Multi-center, randomized, double-blind, parallel-group trial	Sample size: N=514 N=406 (Rotigotine) N= 108 (Placebo) Gender: 285 males Age: 65.4±10.5 DD: N/A advanced stage H&Y (2-4)	OH was found in 3 (3%) in 2mg/24h, 1(1%) in 4mg/24h, 0 in 6mg/24h, 2(2%) in 8mg/24h and 6 (1%) in overall group. OH was present in 7 (6%) placebo treated subjects. No significant difference.		Nicholas, A. P. (2014) ^9^

d=day; DAs= Dopamine receptor agonists; m=month, N/A= not available, OH: orthostatic hypotension, w=week.

Dopamine receptor agonists (DAs): Non-ergot DAs, such as pramipexole, rotigotine and ropinirole, is another large group of drugs which are often used to control the motor and non-motor symptoms in PD patients. DAs was considered having the possibility to exacerbate OH by stimulating baroreceptors and causing peripheral venous and arteriolar dilatation [37, 38].

Pramipexole,one of the wildly used Dopamine receptor agonists in clinic, was reported to worse OH. In Pramipexole On Underlying Disease (PROUD) study, 535 PD patients were included and 261 received pramipexole (1.5mg/d). After 6-15 moths’ treatment, 2 patients developed drug-related postural hypotension [39]. However, study from J P Hubble had drawn a opposite conclusion. In this study, pramipexole was applied in early Parkinson’s patients with an ascending dose of 4.5 mg/day maximum. After treatment with 9 weeks’ duration, all subjects in both the pramipexole and the placebo groups experienced one or more episodes of asymptomatic OH and there was no significant difference between the pramipexole and the placebo groups [40].

Ropinirole, another dopamine receptor agonist, has also been reported to be at risk of orthostatic hypotension. In a study from Titlic M, et al, 50 PD patients were included and one female patient was reported to have OH after the administration of ropinirole [41]. In another multi-center, randomized, double-blind, parallel-group trial, 221 subjects were included and 20.34% cases were found with OH after 12 weeks’ treatment with ropinirole [42].

Rotigotine transdermal system was often applied in patients with advanced PD. Of 90 patients with advanced PD who received rotigotine 8mg/24h for 8 weeks as well as levodopa and oral DA (pramipexole </=1.5 mg/day, ropinirole </=6.0 mg/day), 9 (10%) was reported with OH [43]. In another study, the dose-response relationship between rotigotine and OH was evaluated. In this research, 406 subjects received Rotigotine and 108 received Placebo. Among them, OH was present in 7 (6%) placebo treated subjects. In groups treated with different dosage of Rotigotine, OH was found in 3 (3%) in 2mg/24h, 1(1%) in 4mg/24h, 0 in 6mg/24h, 2(2%) in 8mg/24h and 6 (1%) in overall group. No significant difference was noted between placebo and rotigotine in terms of incidence rate of OH. Similarly, it seems that the increase of rotigotine dose does not significantly increase the incidence of OH[44].

From all the research above, it seems that the PD patient’s age, disease duration, disease severity as well as drug dosage may all related to the occurrence of antiparkinsonian drugs- induced OH.

#### 1.2.2 Neurogenic mechanisms

Cardiac and extra-cardiac sympathetic noradrenergic denervation and baroreflex failure existed in virtually all PD patients with OH detected by neuroimaging, neurochemical, and neuropharmacological studies [45-48]. These three components constituted the so-called “triple whammy” [5]. The cooperation of these three factors contributed to the occurrence of OH [49].

##### 1.2.2.1 Cardiac noradrenergic denervation

More than one study confirms that PD patients have a loss of sympathetic innervation of the heart, as demonstrated by low myocardial concentrations of radioactivity after injection of the sympathoneural imaging agents, 123I-metaiodobenzylguanidine or 6-[18F] fluorodopamine [48-51]. Cardiac sympathetic denervation can occur in the early stage of PD, which is in accordance with brake stage[52, 53]. A patient with primary chronic autonomic failure underwent biennial brain 6-[18F] DOPA and myocardial 6-[18F] dopamine scanning over 4 years and low 6 - [18F] DOPA derived radioactivity in heart occurred 2 years earlier than that in striatum [52], indicating that CAD was an early clinical manifestation in PD. Cardiac noradrenergic denervation can decrease heart rate and cardiac output. Although cardiac noradrenergic denervation was more prevalent in patient with OH, the relationship between the degree of cardiac sympathetic noradrenergic denervation and the severity of OH was uncertain yet. Senard JM found that the degree of cardiac sympathetic noradrenergic denervation correlates with the severity of OH [54]. In contract, Guillaume Lamotte concluded that cardiac noradrenergic deficiency did not predict the severity of OH, a greater role of baroreflex-sympathoneural failure than of cardiac noradrenergic deficiency in determining the magnitude of OH [55].

##### 1.2.2.2 Extra-cardiac noradrenergic denervation

Besides cardiac noradrenergic innervation, the extra cardiac noradrenergic innervation is also related to the occurrence of OH in PD patients. Study found that the decrease of 6-[18F] fluorodopamine-derived radioactivity was most prominent in the heart but was also detectable in extracardiac organs, such as renal cortex, and thyroid [52]. Extra-cardiac sympathetic denervation leads to a decrease in the release of norepinephrine, resulting in decreased peripheral vasoconstriction, reduced return heart blood volume, and eventual decreased BP.

##### 1.2.2.3 Decreased baroreflex response and sympathetic reflex

Cardiac denervation alone will not lead to OH in PD patients. Baroreflex response and sympathetic reflex, which buffers blood pressure against excessive fluctuations, plays critical role in the occurrences of OH in PD patients. Baroreflex circulation consists of afferent and efferent pathways. Through the afferent pathway, the information of vascular pressure is transmitted from the baroreceptors of arteries and veins to the nucleus tractus solitarius (NTS) in the dorsal medulla oblongata. The efferent pathways are mainly composed by two parts: vagal and adrenergic pathway. The vagal baroreflex pathway runs from the NTS to the nucleus ambiguus (NA) and sends efferent signal to the sinoatrial node (SA), which normally controls the activation of the heart and is closely related to heart rate and rhythm. The adrenergic pathway runs from the NTS to the caudal ventrolateral medulla (CVLM), rostral ventrolateral medulla (RVLM), intermediolateral thoracic spinal cord, autonomic ganglia and to the heart, skeletal muscle and splanchnic vessels, ultimately affecting the total peripheral resistance, cardiac output and the control of arterial pressure.

A change of position from supine to an upright position leads to nearly 25-30% of total blood volume accumulated in the skeletal muscle and splanchnic vessels. PD patients mainly involve the nucleus tractus solitarius, rostral ventrolateral medulla, caudal ventrolateral medulla and the sympathetic efferent pathway, which is also known as the central injury mechanism [56]. PD patients with OH often show decreased sympathetic reflex during standing, which fails to correct the decrease in venous return during standing [5, 45, 55, 57-61], leading to a large decrease in BP.

### 1.3 Treatment

The ideal treatment strategy for OH includes three elements: 1) Minimizing the pulse pressure difference related to postural changes; 2) Avoiding supine hypertension as much as possible; 3) Relieving orthostatic hypotension associated symptoms; 4) Decreasing the morbidity and mortality of patients with symptomatic OH. The efficacy and limitation of both Non-pharmacological and pharmacological measures are summarized as follows and some clinical trials are listed in Table 2.

**Table 2:** Drugs proposed for the treatment of orthostatic hypotension.

Mode of action	drugs	Doses	Side effect
Drugs acting on sympathetic pathways	Droxidopa/L-DOPS (eo-3,4-dihydroxyphenylserine)	100-600mg tid	Supine hypertension,headache, dizziness, supine hypertension
	Yohimbine	5.4mg tid	Nausea, tremor, confusion, tension
	Domperidone,	10mg tid	QT prolongation, ventricular tachycardia, sudden cardiac death
	Metoclopramide	-	Nausea, vomiting, loss of appetite, indigestio
	Pyridostigmine	30-60mg tid	Abdominal cramps, nausea, salivation
	Fipamezole	0.1 mg / day	Supine hypertension
Drugs acting on blood vessels	Indomethacin	550 mg/day	Dyspepsia, stomachache, headache, dizziness, kidney damage
	Midodrine	2.5-10mg tid	Abnormal sensation, pruritus, hair erection, supine hypertension
	Ergot alkaloids	1 mg/d	Nausea, vomiting, allergic reactions, severe dyspnea, long-term use can damage vascular endothelial cells
Drugs acting on blood volume	Fludrocortisone	0.1-0.3 mg/d	Hypokalemia, edema, congestive heart failure, supine hypertension
	Erythropoietin	25-75 UI/kg 3 times/week	Supine hypertension
	Desmopressin	-	Headache, nausea, stomachache, hypersensitivity, water retention, hyponatremia
Drugs for postprandial hypotension	Caffeine	100g/d	-
	Octreotide	50-100µg qd	Injection related, abdominal colic, fatigue, headache

#### 1.3.1 Non-pharmacological treatment

Non-pharmacological management is the preferred intervention for OH because it can improve baroreflex sensitivity impairment which is consistently associated with supine hypertension. Non-pharmacological treatment intervenes OH through multiple mechanisms, including ensuring sufficient blood volume, reducing the amplitude and speed of posture change, decreasing the accumulation of blood in peripheral vascular bed, and enhancing baroreflex sensitivity.

##### 1.3.1.1 Enhancing baroreflex sensitivity

Water-drinking treatment is an effective way to enhance baroreflex sensitivity. Drinking water of 16 ounces in 3 to 4 minutes is an emergency treatment to raise systolic BP and reduce orthostatic symptoms [62]. Drinking water of 500 ml increased the gain of arterial baroreflex control of muscle sympathetic nerve activity (MSNA) and remained elevated 60 min. Water drinking was also found to significantly and rapidly raises plasma norepinephrine and sympathetic activity [63]. Notably, saline intake had no effect on arterial baroreflex [64] and water drinking only increases blood pressure in patients with autonomic failure [65].

Treadmill gait training was another way to enhance baroreflex sensitivity. A controlled open-label study evaluating the cardiovascular benefits of partial weight supported treadmill gait training in PD showed that the training stabilized the BP, and enhanced baroreflex sensitivity by 80.4% [66].

##### 1.3.1.2 Decrease the accumulation of blood in peripheral vascular bed

About 25-30% of total blood volume was accumulated in the splanchnic-mesenteric capacitance bed, which is a large volume, low-resistance system [67]. Ways to decrease the accumulation of blood in skeletal muscle and splanchnic vessel can alleviate OH.

Waist-high and custom-fitted thigh compression stockings and sequential application of pressure ranging from 30 to 40 mmHg by abdominal binders, may decrease venous pooling in the lower body [68-70]. Unfortunately, it is difficult to put on for PD patients with disability of independent living and may be uncomfortable in a hot environment.

Physical counter-maneuvers may cut down blood accumulation in lower limbs and prevent standing BP from falling to fatal levels or prevent the occurrence of syncope [71, 72]. However, because of the risk of falls, the last counter-maneuvers should be applied to PD patients presenting with dysfunctional postural reflexes.

##### 1.3.1.3 Others

PD patients with OH should avoid some situations that may cause OH, including a sudden change in position, staying in bed for a long time, standing for a long time, high-temperature environments, excessive intake of alcohol, low salt diets, insufficient fluid intake, high-glycemic index carbohydrates intake [73, 74]. In PD patients, elevation of the head by 6 to 9 inches can reduce stress diuresis at night and reduce decreased blood volume [75, 76]. PD patients with OH should reduce or stop some drugs, which have side effects of causing or exacerbating OH, such as vasodilators, diuretics, sympathetic drugs and alpha-1 blockers [77-79].

#### 1.3.2 Pharmacological treatment

When non-pharmacological methods are unable to effectively control OH, it is necessary to adopt pharmacological treatment, which is regarded as a second-line treatment strategy because they may cause or aggravate supine hypertension [80]. However, it is an indispensable therapeutic regimen for the treatment of OH, if well used, will greatly enhance the therapeutic effectiveness.

##### 1.3.2.1 Noradrenergic and adrenergic pathway

Norepinephrine replacement therapy is more effective in PD patients with neurodegeneration of peripheral noradrenergic fibers. Midodrine, droxidopa and metoclopramide are representative drugs acting on noradrenergic and adrenergic pathway.

###### Midodrine

Midodrine exerts its actions via activation of the alpha1-adrenergic receptors of the arteriolar and venous vasculature, enhancing vascular tone. Midodrine has long been used in clinic and approved for treatment of OH by US Food and Drug and in some European countries [81]. In 1981, it was firstly reported to be a new agent in the management of OH by Schirger. In this study, 5 patients were enrolled and midodrine in a dosage of 2.5 to 5 mg three times daily was found to improve standing blood pressures at the end of 1 week of therapy in all patients. Supine systolic hypertension was reported in two patients [82]. However, another study found that midodrine was not always effective in the treatment of OH. In this double-blind crossover trial, 7 patients were enrolled, and 4 cases were found that arterial pressure decreased during midodrine treatment. Midodrine was only effective in patients with preservation of autonomic reflexes. Conversely, in patients with impaired baroreceptor response, midodrine might exacerbate OH due to extracellular fluid volume depletion [83]. To further evaluate the efficacy and side effects of midodrine in the treatment of OH, in 1997, a twenty-five centers, randomized, placebo-controlled trail was done by Low, P A and his colleague and totally 197 patients were enrolled. In this study, 10-mg dose of midodrine was found to be effective in improvements in standing systolic BP and in the global symptom relief score. The main adverse effects included pilomotor reactions, urinary retention, and supine hypertension [84]. Then in 1998, a dose-response study was done by Wright, R A and his colleague. They found both 10-mg and 20 mg dose of midodrine were effective in the treatment of OH and there was a significant linear relation between midodrine dosage and systolic blood pressure [85]. There is no specific research on the effects of midodrine in PD patients with OH. However, results from two large placebo-controlled studies involving PD patients with OH [84, 86] showed that the patients responded to midodrine treatment.

###### Droxidopa

Droxidopa (L-threo-3,4-dihydroxyphenylserine) is a norepinephrine prodrug which increases the levels of norepinephrine in postganglionic sympathetic neurons and has been used in PD patients with OH since 1989 [87, 88]. Droxidopa is well-tolerated, and the predominant side effect, including supine hypertension, has very low prevalence [89]. A multicenter, randomized, double-blind, placebo-controlled trial of droxidopa for OH in PD showed that droxidopa alleviated mean standing systolic blood-pressure change by 12.5 mmHg. Compared with placebo, droxidopa reduced the incidence of falls and fall-related injuries by about 50% [90]. Another randomized controlled trial showed that droxidopa can significantly increase upright BP by 11.2 mm Hg, resolve nOH-related features of dizziness and lightheadedness, improve levels of daily living. At the meantime, the supine systolic BP increased by 7.6 mm Hg in droxidopa treated patients [90-96]. To further evaluate the effect of droxidopa on fall risk in PD patients with orthostatic hypotension, a 10-week, phase 3, randomized, placebo-controlled, double-blind trial was done by Hauser, R. A. et al in 2016. In this study, a total of 225 patients were randomized. The results showed that the fall rate was 0.4 falls per patient-week in the droxidopa group and 1.05 falls per patient-week in the placebo group. Hauser, Robert A and his colleagues examined the efficacy and tolerability of droxidopa in patients with PD and nOH by using integrated clinical trial data. A total of 307 patients with PD were subtracted for analyzing from 3 phase 3 trail. They found that droxidopa significant increased standing mean systolic/diastolic BP and was well tolerated in patients with PD accompanied with nOH [97].

###### Metoclopramide or domperidone

Metoclopramide or domperidone has been reported to be a potential OH intervention as a dopamine receptor antagonist, because presynaptic dopamine receptors of sympathetic nerve endings contribute to the release of norepinephrine [98, 99]. Some data suggest that domperidone prevents the fall of BP and the frequency of OH caused by dopamine agonists [100]. A systematic review showed that domperidone may improve OH in patients with PD induced by dopaminergic drugs [101]. Domperidone may lead to prolonged QT and increase sudden cardiac death as well as ventricular tachycardia in patients with PD living with underlying heart conditions, so use of domperidone in PD is limited.

###### Fipamezole

Fipamezole, an α2-adrenergic receptor antagonist, can bind to α-adrenergic 1A and 1B receptors, dopamine and norepinephrine transporter [102]. A previous study evaluated acute hemodynamic benefits of fipamezole in 21 PD patients [77]. Acute intravenous levodopa treatment reduced the mean BP significantly. Fipamezole returned BP to preinfusion levels in a dose-dependent fashion.

##### 1.3.2.2 Enhancing residual sympathetic tone therapy

Enhancing residual sympathetic tone is another way to prevent OH. Representative drugs are pyridostigmine and atomoxetine. This treatment is suitable for patients with spared postganglionic fibers.

###### Pyridostigmine

Pyridostigmine, which is an acetylcholinesterase inhibitor, is found to enhance standing blood pressure in patient with OH by inhibiting acetylcholinesterase, augmenting sympathetic traffic at the level of the autonomic ganglia (as preganglionic sympathetic neurons are cholinergic), and enhancing baroreflex function. Due to its novel mechanism, pyridostigmine had no effect on supine hypertension [103]. To further evaluate the effect of pyridostigmine on the treatment of OH in PD patients, a double-center, double-blind, randomized, active-control, crossover, phase II non-inferiority trial of pyridostigmine bromide for OH in PD was done. In this study, nine participants completing each trial arm. Results showed that supine systolic BP increased from 128.4 to 130.4 mmHg after the administration of pyridostigmine [104]. No supine hypertension was found.

###### Atomoxetine

In recent years, the advantages of atomoxetine in the treatment of OH have been concerned by scholars [105-110]. Atomoxetine, which is a norepinephrine transporter blockade, can significantly increase SBP, improving upright blood pressure and orthostatic hypotension-related symptoms in autonomic failure patients with residual sympathetic activity. In a comparative study, atomoxetine was superior to midodrine in reducing SBP and improving OH- related symptoms [110]. To further evaluate the efficacy and safety of atomoxetine, a prospective open-label randomized trial was done. In this study, 50 patients with symptomatic neurogenic orthostatic hypotension (nOH) were included and randomly assigned to atomoxetine and midodrine group. The subjects received either atomoxetine 18 mg daily or midodrine 5 mg twice daily. Results after 1 month treatment, both atomoxetine and midodrine enhance the standing BP in almost 74% subjects, whereas only atomoxetine resulted in significant symptomatic improvements [105]. Three (3/50, 6%) of the patients who were reevaluated at 1 month reported adverse events, among them, two had frequent sweating, and one had frequent urination. The adverse effects are slight [105].

##### 1.3.2.3 Others

Flucortisone

Flucortisone reduces the deficiency of plasma renin and aldosterone when standing as a synthetic mineralocorticoid with little glucocorticoid activity, so it can be used to treat OH. Studies have shown that low-dose flucortisone (0.1 mg/day) improve OH combined with a high pillow position and a high-salt diet at night [111, 112]. However, because flucortisone has a long action time and continues to raise BP, it is not suitable for controlling nighttime BP. It should be noted that the use of mineralocorticoid leads to the increased mortality and risk of hypokalemia. In a comparative study, pyridostigmine is superior to flucortison since more effective in enhancing OH and less side effects on supine hypertension were found in pyridostigmine group.

###### Indomethacin

Indomethacin is a nonsteroidal drug with anti-inflammatory and antipyretic properties. The mechanism of indomethacin on OH intervention is unclear. Indomethacin may reduce the drop in BP via increasing reflex vasoconstriction due to promoting the release of endogenous angiotensin II and noradrenaline from remaining nerve ending [113, 114]. The effect of indomethacin on OH was once evaluated in 6 patients with OH and 5 normal subjects. Mean blood pressure enhanced from 104 to 122 mmHg after the administration of indomethacin [113].

## 2.Supine hypertension

### 2.1 Summary

Supine hypertension (SH) is another CAD in patients with PD and About one half of patients with autonomic failure also suffer from supine hypertension. SH is defined as the systolic BP ≥150 mmHg and the diastolic BP ≥90 mmHg when in the supine position [115]. SH can worse OH through pressure natriuresis. Therefore, OH and SH often coexist in PD with CAD. Supine hypertension in PD cases with OH may complicate the treatment and increase contribution to organ damage and early death [116-119].

### 2.2 Pathogenetic mechanisms

The cause and mechanisms of supine hypertension remain still unknown. Supine hypertension in PD patient with autonomic failure may be mediated by an increase in peripheral vascular resistance due to residual sympathetic tone[120]. In PD with OH, baroreflex impairment was also associated with the supine hypertension[81].

### 2.3 Treatment

The treatment goal of supine hypertension is to regulate dysfunction of target organs and the risk for OH. The indications for beginning the management of supine hypertension are patient’s BP persistence greater than 180/110 mmHg (systolic and diastolic), and the treatment should be individualized for systolic blood pressure (SBP) 160-180 mmHg and diastolic blood pressure (DBP) 90-110 mmHg [88]. Supine hypertension complicates the treatment of OH, which may cause stressful diuresis at night and aggravate the symptoms of OH in the morning. Therefore, OH and the risk of falling should also be considered when those antihypertensive drugs are used to treat supine hypertension.

#### 2.3.1 Non-pharmacologic therapy

There is need to create educate patients with supine hypertension that they should not stay in supine position when taking anti-hypotensive drugs or using compression stockings or abdominal belts. One recent research also showed that passive heating of the pelvis and abdomen with a commercial heating pad can reduce BP in patients with supine hypertension and autonomic nervous failure [121].

#### 2.3.2 Pharmacological treatment

When non-pharmacological treatment cannot be effective, pharmacological interventions may be considered to be used in supine hypertension. Because supine hypertension is often combined with OH, it is clinically difficult to treat supine hypertension, and antihypertensive drugs should be able to control BP overnight, while not causing OH in the morning. Table 3 describes the mode of action, dosage and side effects of some drugs used to treat supine hypertension.

##### Clonidine

Clonidine, as an α2-adrenergic agonist, is a central antihypertensive drug suitable for patients with severe supine hypertension. Residual sympathetic tone acts on the adrenergic receptors during hypertension, but it is a possible mechanism for PD patients to develop supine hypertension, allowing clonidine to be used for severe supine hypertension. However, BP should be closely monitored during this time [120].

Short-acting nifedipine and nitroglycerin transdermal patches

Nitroglycerin transdermal patches are highly maneuverable, but their antihypertensive effects may be unstable [122]. The low-dose sustained-release preparation of the nitroglycerin transdermal patch (0.1-0.2 mg/h) lead to hypotension within 24 hours [123]. Because nitroglycerin has a short action time, BP can be stopped quickly by removing the patch, which helps reduce the occurrence of OH in the morning [80, 123]. When using nitroglycerin transdermal patches, patients should be advised not to get up at night to avoid hypotension and fainting. If patients want to get up to the toilet, it is best to remove the patch before getting up.

Nitroglycerin transdermal patch and nifedipine lower BP, but these two drugs have no effect on stressful diuresis at night [124]. Nifedipine is a more effective antihypertensive drug, and initial treatment should be 10 mg before going to bed [80, 125]. However, nifedipine can increase urine sodium excretion and worsen OH [124, 126]. In essential hypertension, these two drugs are not the first choice, and can reflexively activate sympathetic nerves and renin, reducing survival rates [126, 127]. These effects are not present due to decreased baseline renin activity and baseline sympathetic activity of patients with autonomic failure; making them unable to effectively modulate baroreflexes.

**Table 3:** Drugs proposed for the treatment of Supine Hypertension.

Mode of action	Medications	Doses	Side effect
α- 2 adrenergic agonists	Clonidine	0.1mg evening	dry mouth, bradycardia, atrophy
Release nitric oxide	Nitroglycerin transdermal patch	0.1-0.2mg/hr applied at bedtime, remove in the morning	hypotension and fall risk
Calcium antagonist	short acting nifedipine	10-30mg bedtime	OH
Selective β1-adrenergic receptor blocker	Nebivolol	2.5-5 mg bedtime	-
Selective MR antagonist	Eplerenone	50mg bedtime	-
AT1 receptor blocker	Losartan	50mg bedtime	OH
Block the degradation of cyclic guanosine monophosphate	Sildenafil citrate	50mg bedtime	-

AT: angiotensin II receptor blocker; MR: mineralocorticoid receptor; OH: Orthostatic hypotension.

##### Sildenafil citrate

Sildenafil citrate enhances the function of nitric oxide as an effective vasodilator [128, 129]. In 8 supine patients with nOH and hypertension, the maximum reduction in systolic blood pressure was 30 mmHg in the sildenafil group approximately 8 hours after 25 mg of sildenafil compared with placebo [130]. Sildenafil is not recommended to be taken with a nitric oxide donor. Sildenafil also did not affect sodium excretion in patients with autonomic nervous failure, and acute use did not have adverse effects.

##### Nebivolol

Nebivolol, a third-generation selective β1-adrenergic receptor blocker, has unique vasodilatory actions which is dependent on increasing the bioavailability of endothelium derived nitric oxide. Although nebivolol has a strong effect on lowering BP, it does not worsen OH in the morning. One study showed that nebivolol significantly reduced nocturnal BP (about 40 mmHg) in patients exhibiting good response to 25 mg sildenafil citrate [131]. Nebivolol is well-tolerated, and the recommended starting treatment with nebivolol is 2.5 mg in the evening.

##### Eplerenone

Eplerenone (at 50mg), as a selective mineralocorticoid receptor antagonist, has been showed to reduce maximum BP by about 32mmHg in supine hypertension [132]. The mechanism of eplerenone is not related to diuresis and does not increase urinary sodium excretion so that it does not deteriorate OH in the morning. The possible mechanisms for lowering BP include the blockade of hormone-independent ligands, angiotensin II, aldosterone, or cortisol [132-134], but further research is needed to determine whether long-term use of the drug worsens OH in the morning.

##### Losartan

Losartan, an At_1_ receptor blocker (ARB), decreased fluid loss and pressure natriuresis. After taking 50 mg losartan for 6 hours, the BP of 11 patients with severe autonomic nervous failure decreased by 32 mmHg, what’s more, losartan had minimal effects on morning NOH [135]. However, ARB treatment showed the following benefits: improved cerebral autoregulation in patients with stroke having diabetes and hypertension [136] [137]. To avoid drug interactions, PD patients taking levodopa and benzimidazole should choose angiotensin II At_1_ receptor antagonists, angiotensin converting enzyme inhibitors, or β-adrenocortical hormone as the first choice of antihypertensive drugs [77].

## 3.Postprandial hypotension

Postprandial hypotension is considered among the earliest events of CAD in PD. Postprandial hypotension may be misdiagnosed as a state of dyskinesia, accompanied by worsening stiffness and bradykinesia [138-140]. PD patients with OH often experience postprandial hypotension because these individuals’ impaired autonomic reflexes cannot be compensated for splanchnic blood pooling following eating. The drop degree of BP depends on the amount of food ingested [141] and its contents, especially carbohydrate content [142, 143]. The hypotensive effect of a meal develops within 15 minutes, reaching its maximum and persisting up to 3 hours after the start of a meal [144, 145]. Some possible mechanisms of postprandial hypotension include dysfunctional sympathetic reflexes in response to visceral r vasodilation and / or obstruction of insulin with the normal sympathetic nervous system [146, 147]. Two studies have shown that vasoactive gastrointestinal peptides are interrelated to the postprandial hypotension [148, 149]. Nonpharmacologic approaches are a very cost-effective treatment strategy for patients with postprandial hypotension. Patients should eat small and frequent meals, reduce carbohydrate intake or consume high-carbohydrate foods before going to bed, while paying attention to the risk of low blood sugar and high blood sugar at night. Caffeine and water can reduce symptoms of postprandial hypotension. The diuretic effect of caffeine may counteract its vasopressor benefits [150]. A systematic review suggests that octreotide and acarbose (moderate evidence, strong recommendation) or caffeine or voglibose should be applied in cases of severe postprandial hypotension [104, 151].

### Yohimbine

Yohimbine which activates adrenergic receptors and promotes the production of norepinephrine from peripheral sympathetic nerve endings [4, 152]. Yohimbine is a weaker agonist of α2 receptor and a slight effect on the α1 receptor. Yohimbine is preferred for postprandial hypotension [152].

## 4. Nondipping

Dipping" refers to a 10-20% decrease in BP at night [153]. Nondipping is on the other hand considered a nocturnal BP decreasing below 10% [154-156]. Several definitions have been proposed in various studies hence a more standardized definition should be investigated in relation to BP cut-off values, mean arterial pressure (MAP) among others. Theoretically, the diagnosis of a 10% decrease in nocturnal MAP is proposed [157]. Currently, there are few reports on nondipping in PD. Reverse dipping may reflect autonomic dysfunction in PD, implying the presence of nocturnal BP dysregulation in PD patients [155, 158]. A "nondipping" mode may contribute to heart and other organs injuries. However, currently there is no clear data on the treatment of nondipping. It is not clear whether antihypertensive agents can convert nondipping BP pattern into a dipping pattern [155, 157, 159].

## 5. Conclusion

PD is a common cause of autonomic dysfunction [160-162], and there are a series of complex disorders of BP control mechanisms including OH, postprandial hypotension, supine hypertension, and nondipping. These disorders can be caused by different mechanisms, but they may all be related to baroreflex disorders. The goal of treatment is to reduce the occurrence of related symptoms and the decline of quality of life caused by abnormal BP control disorders, rather than simply regulating the value of blood pressure. Non-drug treatment methods are preferred. If the effects are not adequate, drugs can be added. In the future, a series of studies are needed to further understand the development mechanism and produce more effective treatment strategies for CAD in PD.

## Supplementary Materials


