# Supplementary material for: The Pathogenesis and Treatment of Cardiovascular Autonomic Dysfunction in Parkinson’s Disease: What We Know and Where to Go
Source: Aging Dis. 2021 Oct 1;12(7):1675–92. doi: 10.14336/AD.2021.0214 (PMC8460297; doi:10.14336/AD.2021.0214)
Supplement: Supplementary file 1 [file AD-12-7-1675-s.pdf]

SUPPLEMENTARY DATA

The Diagnosis and Treatment of Cardiovascular Autonomic Dysfunction in Parkinson's Disease

Shuzhen Zhu[#,*], Xiaoyan Xu[#], Yuqi Luo[#], Bin Deng, Xingfang Guo, Yang Guo, Aimin Chen, Xiaobo Wei, Qing Wang[*]

© 2021. Zhu S et al. Published online at http://www.aginganddisease.org/EN/10.14336/AD.2021.0214

SUPPLEMENTARY DATA

Supplementary Table 1. Screening questions for suspected OH/NOH [1]

**Screening questions:**
1 Have you fainted/blacked out recently?
2 Do you feel dizzy or lightheaded upon standing?
3 Do you have vision disturbances when standing?
4 Do you have difficulty breathing when standing?
5 Do you have leg buckling or leg weakness when standing?
6 Do you ever experience neck pain or aching when standing?
7 Do the above symptoms improve or disappear when you sit or lay down?
8 Are the above symptoms worse in the morning or after meals?
9 Have you experienced a fall recently?
10 Are there any other symptoms you commonly experience when you stand up or within 3–5 min of standing and get better when you sit or lay down?

1. Gibbons CH, Schmidt P, Biaggioni I, et al. The recommendations of a consensus panel for the screening, diagnosis, and treatment of neurogenic orthostatic hypotension and associated supine hypertension. *J Neurol*. Aug 2017;264(8):1567-1582. doi:10.1007/s00415-016-8375-x

Supplementary Table 2. Proposed grading scale for NOH [1].

**Grade**	**Attributes**
1	Infrequent symptoms/unrestricted standing time AND mild OH [20-30 mmHg drop in SBP during supine-to-standing test]
2	≥5 min standing time (but not unrestricted) AND [> 30 mmHg drop in SBP OR moderate impact ADL]
3	< 5 min standing time AND [> 30 mmHg drop in SBP OR severe impact on ADL]
4	< 1 min standing time AND [> 30 mmHg drop in SBP OR incapacitated]

A patient with grade 3 or 4 NOH should be treated by a healthcare provider with experience in managing NOH
SBP systolic blood pressure, ADL activities of daily living
[1] Low PA (2015). Neurogenic orthostatic hypotension: pathophysiology and diagnosis. Am J Manag Care, 21:s248-257.

SUPPLEMENTARY DATA

Supplementary Table 3. Orthostatic intolerance [1].

Question on orthostatic NO.	Question	Response code<br>0	1	2	3	4	5<br>5	6<br>6	7<br>7
1	In the past year, have you ever felt faint, dizzy, "goofy", or had difficulty thinking soon after standing up from a sitting or lying position? (Response: 1= yes, 2= no)		1	0					
2	When standing up, how frequently do you get these feelings or symptoms? (Response: 1= rarely, 2= occasionally, 3= frequently, 4= almost always)		0	1	2	3			
3	How would you rate the severity of these feelings or symptoms? (Response:1= mild, 2= moderate, 3= severe)		1	2	3				
4	In the past year, have these feelings or symptoms that you have experienced: (Response: 1= gotten much worse, 2= gotten somewhat worse, 3= stayed about the same, 4= gotten somewhat better, 5= gotten much better, 6= completely gone)		3<br>2	1	1	0	0	0	

1. Sletten DM, Suarez GA, Low PA, Mandrekar J, Singer W. COMPASS 31: a refined and abbreviated Composite Autonomic Symptom Score. *Mayo Clin Proc*. Dec 2012;87(12):1196-201. doi:10.1016/j.mayocp.2012.10.013

Supplementary Table 4. Orthostatic hypotension questionnaire[1]

AUTONOMIC DYFUNCTION SCORES ORTHOSTATIC HYPOTENSION QUESTIONNAIRE (OHQ)

Patient Instructions:
We are interested in measuring the symptoms that occur because of your problem with low blood pressure (orthostatic hypotension) and the degree that those symptoms may interfere with your daily activity. It is important that we measure the symptoms that are due ONLY to your low blood pressure, and not something else (like diabetes or Parkinson's disease). Many people know which of their symptoms are due to low blood pressure. Some people who have recently developed problems with low blood pressure may not easily distinguish symptoms of low blood pressure from symptoms caused by other conditions. In general, symptoms of your low blood pressure problem will appear either upon standing or after you have been standing for some time and will usually improve if you sit down or lie down. Some patients even have symptoms when they are sitting which might improve after lying down. Some people have symptoms that improve only after sitting or lying down for quite some time.
Please answer the questions on the following pages keeping in mind that we want to know only about those symptoms that are from your problem with low blood pressure.

OH SYMPTOM ASSESSMENT (OHSA)
Please tick the number on the scale that best rates how severe your symptoms from low blood pressure have been on the average over the past week. You should respond to every symptom. If you do not experience the symptom, circle zero (0).
YOU SHOULD RATE ONLY THE SYMPTOMS THAT ARE DUE TO YOUR LOW BLOOD PRESSURE PROBLEM.

1. Dizziness, lightheadedness, feeling faint, or feeling like you might black out
None □0 □1 □2 □3 □4 □5 □6 □7 □8 □9 □10 Worst possible

2. Problems with vision (blurring, seeing spots, tunnel vision, etc.)
None □0 □1 □2 □3 □4 □5 □6 □7 □8 □9 □10 Worst possible

3. Weakness
None □0 □1 □2 □3 □4 □5 □6 □7 □8 □9 □10 Worst possible

4. Fatigue
None □0 □1 □2 □3 □4 □5 □6 □7 □8 □9 □10 Worst possible

5. Trouble concentrating
None □0 □1 □2 □3 □4 □5 □6 □7 □8 □9 □10 Worst possible

6. Head/neck discomfort
None □0 □1 □2 □3 □4 □5 □6 □7 □8 □9 □10 Worst possible

OH DAILY ACTIVITY SCALE (OHDAS)
We are interested in how the low blood pressure symptoms that you experiences affect daily life. Please rate each item by ticking the number that best represents how much on the average the activity has been interfered with over the past week by the low blood pressure symptoms you have experienced.
If you cannot do the activity for reasons other than low blood pressure, please check the box at right.

SUPPLEMENTARY DATA

1. Activities that require standing for a short time Cannot do for other reasons
No Complete Cannot do for
interference □0 □1 □2 □3 □4 □5 □6 □7 □8 □9 □10 interference □ other reasons

2. Activities that require standing for a long time
No Complete
interference □0 □1 □2 □3 □4 □5 □6 □7 □8 □9 □10 interference □

3. Activities that require walking for a short time
No Complete
interference □0 □1 □2 □3 □4 □5 □6 □7 □8 □9 □10 interference □

4. Activities that require walking for a long time
No Complete
interference □0 □1 □2 □3 □4 □5 □6 □7 □8 □9 □10 interference □

1. Kaufmann H, Malamut R, Norcliffe-Kaufmann L, Rosa K, Freeman R. The Orthostatic Hypotension Questionnaire (OHQ): validation of a novel symptom assessment scale. *Clin Auton Res*. Apr 2012;22(2):79-90. doi:10.1007/s10286-011-0146-2

© 2021. Zhu S et al. Published online at http://www.aginganddisease.org/EN/10.14336/AD.2021.0214
